# Colloidal Interactions in Simulated Intestinal Fluids: Implications for Oral Drug Delivery at the Nanoscale

**DOI:** 10.1002/smsc.202500350

**Published:** 2025-08-12

**Authors:** Juliane Fjelrad Christfort, Matteo Tollemeto, Yudong Li, Lasse Højlund Eklund Thamdrup, Jan van Hest, Anja Boisen

**Affiliations:** ^1^ The Danish National Research Foundation and Villum Foundation's Center IDUN Department of Health Technology Technical University of Denmark 2800 Kgs. Lyngby Denmark; ^2^ Institute for Complex Molecular Systems Department of Biomedical Engineering Eindhoven University of Technology 5600 Eindhoven The Netherlands

**Keywords:** bile salts, chitosan, mesoporous silica nanoparticles, micelles, mucoadhesion, polymersomes, solubilities

## Abstract

Oral drug delivery remains the most preferred administration route, and new oral delivery concepts continuously arise to enable oral delivery of new therapeutics. This study investigates how colloidal structures in five simulated intestinal fluids (SIFs) with varying bile salt and phospholipid compositions influence drug solubility, nanoparticle aggregation and cytotoxicity, and mucoadhesion of nanoparticles and polymers. For the poorly water‐soluble drugs indomethacin and felodipine, colloidal structure size in SIFs varies with solubility, and felodipine's solubility is influenced by the lipid composition. Nanoparticles, including polymersomes and mesoporous silica nanoparticles with different surface charges, are characterized in each medium. Dynamic light scattering reveals three interaction modalities: interaction, aggregation, and combination, depending on nanoparticle type and fluid composition. Additionally, interaction patterns correlate with Caco‐2 cell cytotoxicity. Quartz crystal microbalance with dissipation analysis reveals that both particle and polymer interactions with mucin are significantly altered in SIFs. For nanoparticles, mucin interactions differ depending on the type of nanoparticle. For the polymers, polyethylene oxide completely loses mucin interaction in SIFs, while chitosan retains partial mucoadhesion. These findings emphasize the importance of not only studying drug properties, but also cell compatibility and mucoadhesion of polymers and nanoparticles, in physiologically relevant conditions.

## Graphical abstract

The interactions between colloidal structures in intestinal fluids and therapeutics, nanoparticles, and polymers are investigated. By designing different simulated intestinal fluids, reveal how the concentration and type of bile salts and lipids affect drug solubility, nanoparticle aggregation and cytotoxicity, as well as mucoadhesion of nanoparticles and polymers. All factors that are critical for advancing oral drug delivery.

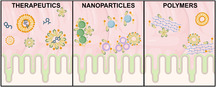

## 1 Introduction

Oral drug delivery remains the most common and preferred administration route due to high patient acceptance and simple manufacturing and distribution processes. Thus, new oral delivery concepts are continuously explored to improve the treatment efficiency of existing oral drugs or enable oral delivery of new or parental drug compounds.^[^ ^1^, ^2^, ^3^ ^]^ In this regard, mucoadhesion is a desired feature, due to the potential of increased intestinal retention, which can improve drug absorption by bringing the drug closer to the epithelial barrier, eventually leading to a reduced dose or lower dosing frequency.^[^ ^4^, ^5^ ^]^ A common strategy to enhance mucus interactions and enable targeted delivery involves the use of nanoparticles, which offer a high surface area per volume and versatile tunability in terms of size, shape, and surface chemistry.^[^ ^3^, ^6^, ^7^ ^]^

**Figure 1:**
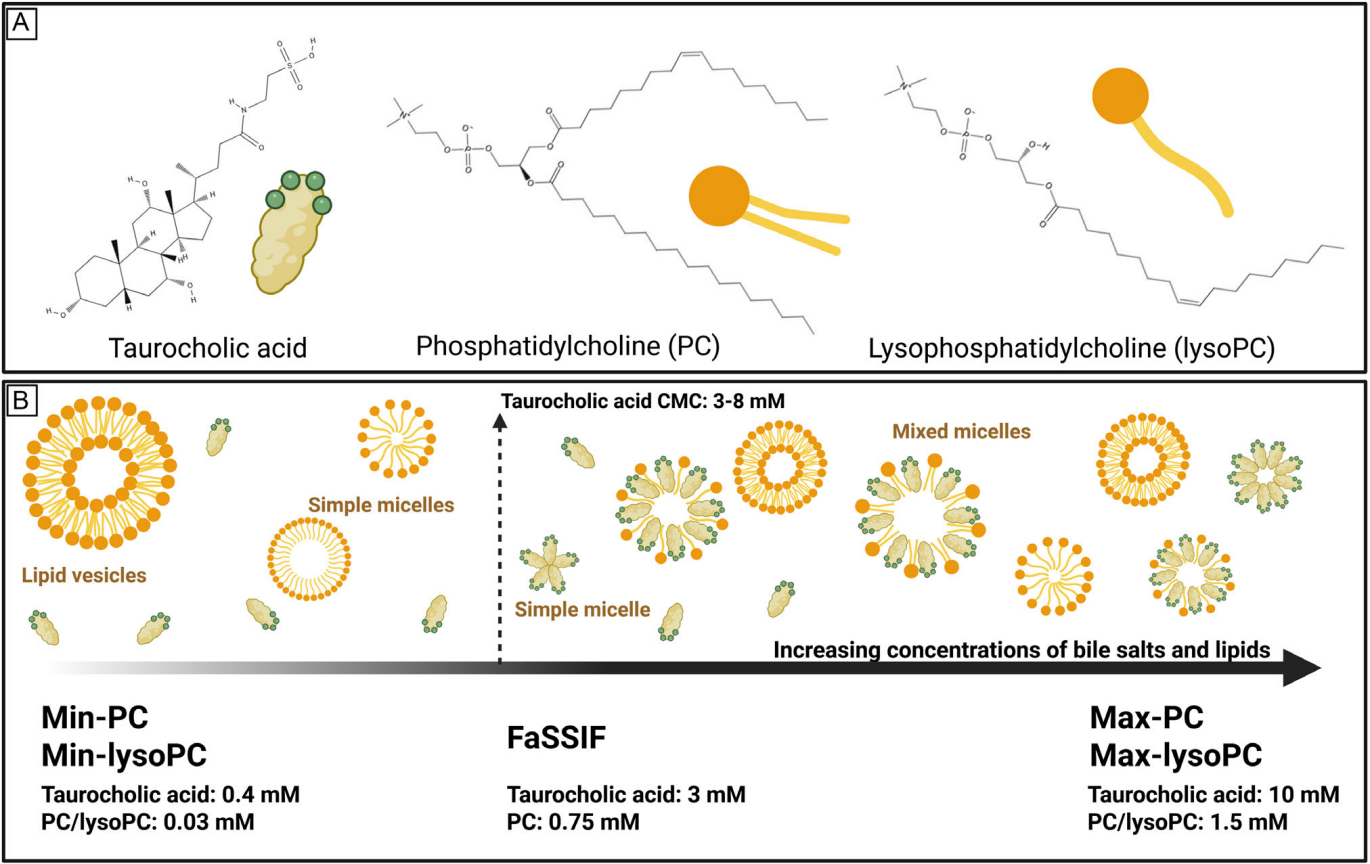
A) Chemical structures of the bile salt taurocholic acid and the phospholipid classes phosphatidylcholine (e.g., 36:1) and lysophosphatidylcholine (e.g., 18:1). B) Schematic overview of the bile salt and phospholipid concentrations in the five simulated intestinal fluids (including the commercially available FaSSIF, Biorelevant), and the expected micellar structures formed. In the two low‐concentration fluids (Min‐PC and Min‐lysoPC), only lipid vesicles and simple micelles of lysoPC are expected. When increasing the concentration of taurocholic acid above the CMC of the bile salt, simple bile salt micelles and mixed bile salt/phospholipid micelles are also expected.

Despite extensive progress in the field of oral drug delivery, it remains challenging to use in vitro data to accurately predict how oral drug delivery systems perform in the gastrointestinal (GI) tract.^[^ ^5^, ^8^, ^9^ ^]^ Following ingestion, oral formulations face complex and varying physicochemical environments and are exposed to heterogeneous biological fluids with changing composition throughout the GI tract.^[^ ^10^, ^11^, ^12^, ^13^ ^]^ The composition varies both based on the location in the GI tract and also on the prandial state of the individual.^[^ ^14^, ^15^, ^16^ ^]^ The effect of pH is one of the most extensively studied and well‐characterized physiological triggers, with drug delivery systems needing to withstand the acidic environment of the stomach before encountering varying pH gradients throughout the intestines. However, the varying presence and concentrations of additional compounds, for example, bile salts, lipids, lipid digestion products, proteins, and enzymes, also affect the fate of drug delivery systems.^[^ ^12^ ^]^ Bile salts and phospholipids are especially interesting, since they facilitate the formation of simple and mixed micelles in water, due to their amphiphilic nature. Despite their structural differences, most phospholipids share the fundamental ability to self‐assemble into lipid vesicles when dispersed in water.^[^ ^7^ ^]^ However, certain types, particularly lyso‐variants or those present in mixed lipid systems, can also form micelles under similar conditions.^[^ ^17^, ^18^ ^]^ In contrast to the relatively small polar head group and the longer hydrophobic tails of phospholipids and other more traditional amphiphiles, bile salts have a flatter structure with a more even distribution of the hydrophilic and the hydrophobic side^[^ ^19^ ^]^ (**Figure** **1**A). Alone, bile salts form simple micelles in water, whereas they also have the ability to convert lipid bilayers into mixed micelles.^[^ ^18^, ^20^ ^]^ Thus, a very common type of colloidal structure in intestinal fluid is mixed micelles formed from bile salts and phospholipids.^[^ ^12^, ^21^, ^22^ ^]^

To bridge the gap between the complex environment in the human GI tract and in vitro testing, simulated GI fluids, also commonly referred to as biorelevant media, have become increasingly popular in preclinical research and development. Simulated GI fluids mimic selected properties of GI fluids in different prandial conditions (e.g., fasted and fed state) and different GI sections (e.g., stomach, small intestine, and colon). Most simulated intestinal fluids, including commercially available fluids, account for selected conditions such as pH, osmolality, and total concentration of bile salts and phospholipids.^[^ ^23^, ^24^ ^]^ Often, one simulated intestinal fluid is applied to reflect average concentrations of bile salts and phospholipids, which comes with the risk of masking variations between low and high concentrations. Additionally, taurocholic acid (in the form of sodium taurocholate) and phosphatidylcholine (PC) (Figure 1A) are often added as substitutes for the numerous different bile salts and lipids in intestinal fluids,^[^ ^20^ ^]^ where less research has focused on the impact of different bile salts or phospholipids, for example, lysophosphatidylcholine (lysoPC) (Figure 1A). With respect to colloidal interactions in GI fluids, lyso forms are especially interesting since they may form micelles instead of vesicles in water.^[^ ^17^ ^]^ Simulated intestinal fluids have been widely used to study how the solubilizing abilities of the amphiphilic compounds in intestinal fluids affect the solubility of poorly water‐soluble drugs^[^ ^25^, ^26^, ^27^ ^]^ and how inherent bile salts affect drug diffusion through mucus.^[^ ^28^ ^]^ However, much less attention has been given to how bile salts and lipids affect the properties of other excipients in oral drug delivery systems, such as polymers.^[^ ^29^, ^30^, ^31^ ^]^ This knowledge gap is especially pronounced for mucoadhesive excipients, where a deeper understanding of how bile salts and lipids affect mucus interactions is still lacking.

This paper explores interactions between colloidal structures in simulated intestinal fluids and various drugs, nanoparticles, and polymers from different perspectives. The concept of “corona formation”, describing how particles interact with biomolecules in physiological solutions, has been extensively studied.^[^ ^32^ ^]^ However, previous research has focused on interactions with proteins in other physiological fluids, such as blood, and not on how particles for oral delivery interact with intestinal fluids.^[^ ^12^, ^32^ ^]^ Similar interactions in the gut remain under‐investigated, and only recently, nonprotein components (such as bile salts and lipids) have been considered.^[^ ^33^ ^]^ The relevance of these interactions can be significant in cases where polymers or nanoparticles for oral delivery rely on specific surface interactions for penetration or adhesion to cells or mucus, which can be hindered by the formation of a biomolecular corona. We study how these GI fluid components affect solubility, cytotoxicity, and mucoadhesion based on a selection of simulated intestinal fluids covering physiologically relevant concentrations of bile salts and phospholipids in the fasted human small intestine,^[^ ^34^, ^35^, ^36^ ^]^ including a commercially available simulated intestinal fluid (biorelevant) (Figure 1B). To be able to extract clear mechanistic information, we chose to focus on simple and well‐defined simulated intestinal fluids mimicking the fasted state. For various types of nanoparticles, we evaluated how simulated intestinal fluids influence their behavior, considering both the materials they are composed of and differences in surface charge. Additionally, we examined how polymers commonly used in oral delivery systems, exhibiting different modes of interaction with mucin, such as electrostatic attraction or hydrogen bonding, are affected by simulated intestinal fluids. Second, we explore if the type of phospholipid affects these properties, by comparing to simulated intestinal fluids prepared with lysoPC instead of PC, but with the same total concentration. Although PC is the preferred group of phospholipids for preparation of simulated intestinal fluids, and the most abundant phospholipid class in secreted bile,^[^ ^37^ ^]^ previous investigations found lysoPC to be the most prevalent phospholipid class in fasted intestinal fluids from humans.^[^ ^38^, ^39^ ^]^ We previously observed the same tendency in intestinal cross‐sections from fasted rats.^[^ ^40^ ^]^ Since lysoPC is the hydrolyzed form of PC, this is in line with the expected enzymatic degradation in the small intestine.

It is highly relevant to study how colloidal structures in intestinal fluid interact with different components in oral drug delivery systems, since this knowledge will allow us to understand in more detail how these interactions modulate their performance in vivo. Based on this knowledge, we can determine which properties or compounds require consideration of micellar interactions and which can be studied using a simple physiological buffer.

## 2 Results and Discussion

### 2.1 Simulated Intestinal Fluids and Interactions with Poorly Water‐Soluble Drugs

**Figure 2:**
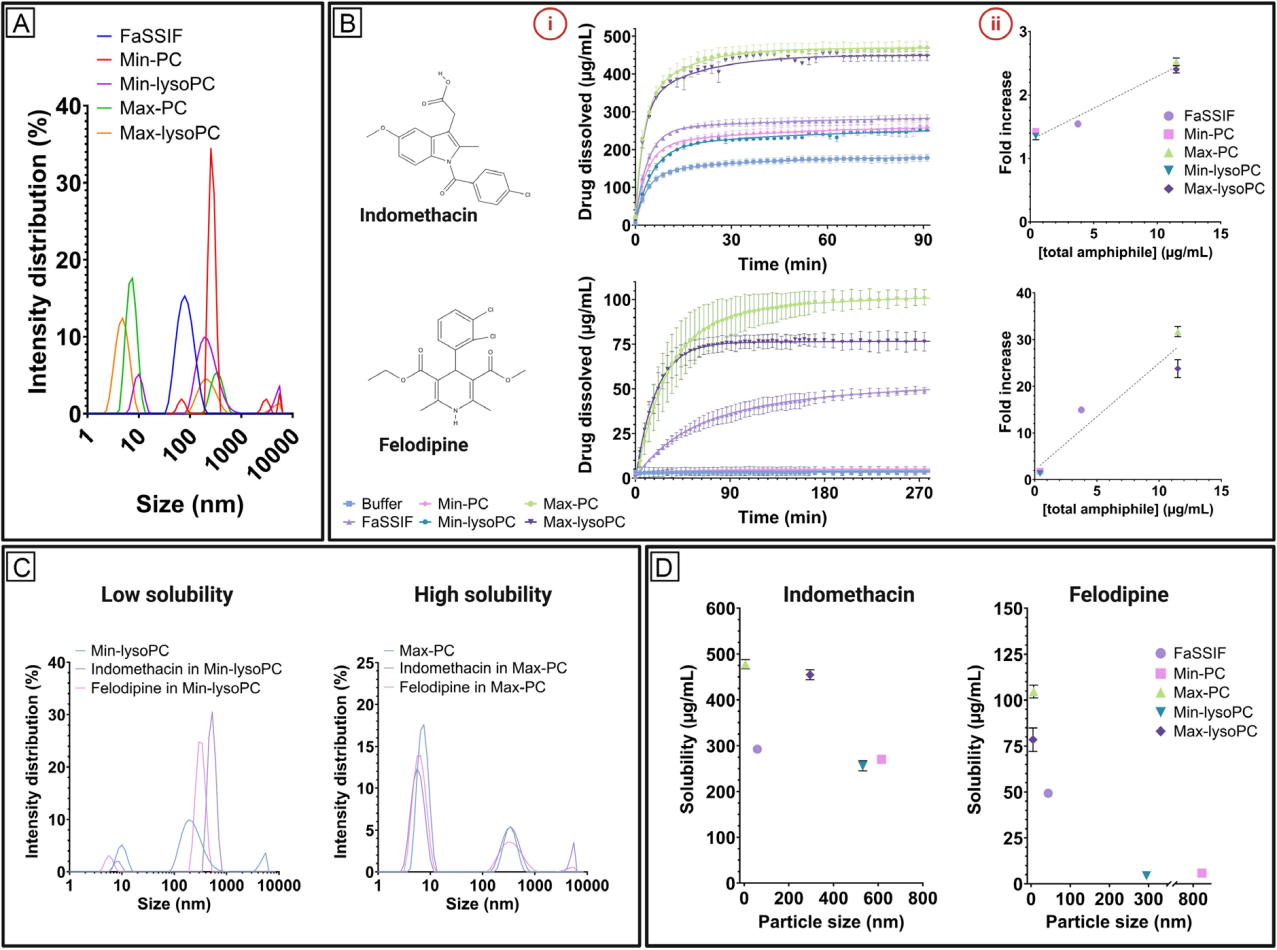
A) Hydrodynamic size distribution of the five simulated intestinal fluids. B) (i) Dissolution and apparent solubility of indomethacin and felodipine, and (ii) the relation between the total concentration of amphiphiles and the increase in solubility compared to the solubility in buffer. C) Hydrodynamic size distribution of indomethacin and felodipine in Min‐lysoPC (left) and Max‐PC (right). D) Hydrodynamic particle size for indomethacin (left) and felodipine (right) as a function of solubility. The error bars show ± SD (*N* = 3‐4).

To evaluate the colloidal size distribution in the five prepared simulated intestinal fluids, we measured the hydrodynamic size using dynamic light scattering (DLS) (**Figure** **2**A). For the commercial fasted state simulated intestinal fluid (FaSSIF), the size distribution was dominated by a single peak representing particles with a diameter of ≈79 nm. For all other simulated intestinal fluids, we observed a multimodal size distribution where the medium size varied between ≈190–340 nm in diameter. This is in accordance with previously reported sizes for mixed micelles formed from bile salts and phospholipids.^[^ ^24^, ^25^ ^]^ For Min‐lysoPC, Max‐PC, and Max‐lysoPC, smaller structures with a size of 5–10 nm in diameter were observed in addition to the larger colloidal structure. For Max‐PC and Max‐lysoPC, this was expected since the concentration of the bile salt was above the critical micelle concentration (CMC) (typically reported to be 3–8 mM for bile salts).^[^ ^25^, ^41^, ^42^ ^]^ Thus, in these conditions, bile salts would be expected to form smaller simple micelles alone. For Min‐lysoPC, on the other hand, the bile salt concentration is well below the CMC, and therefore, we did not expect to observe small colloidal structures. For this simulated intestinal fluid, we assign the small structures to be formed by lysoPC, since smaller phospholipids can be expected to aggregate into simple micelles in water, like bile salts.^[^ ^43^ ^]^ This is supported by the fact that we did not observe any small colloidal structures for Min‐PC, which was prepared with the nonhydrolyzed lipid PC (Figure 2A).

To evaluate the impact of the different simulated intestinal fluids on the solubility of poorly water‐soluble drugs, we investigated the apparent solubility of two drugs, namely indomethacin and felodipine (**Table** **1**), at 37 °C using a microDISS Profiler (Figure 2B). Excess powder (at least 2‐3 times the expected equilibrium solubility) was added to 10 mL preheated simulated intestinal fluids, and the dissolution was monitored until a plateau was reached. As a general trend, both drugs showed the lowest apparent solubility in the buffer without bile salts or phospholipids, and the solubility increased with increasing total concentration of amphiphiles. This is in line with previously reported observations.^[^ ^26^, ^27^ ^]^ Interestingly, a dramatic increase was observed for the apparent solubility of felodipine in FaSSIF (49.3 ± 1.9 μg mL^−1^), where the bile salt concentration reaches the expected CMC (3–8 mm ^[^ ^25^, ^41^, ^42^ ^]^), compared to Min‐PC and Min‐lysoPC (5.9 ± 0.4 and 4.4 ± 1.9 μg mL^−1^, respectively), where the concentration is below the CMC. This is likely because of the neutral and more lipophilic properties of felodipine (Table 1), where it is expected to see a larger benefit of the solubilization properties of the formed micelles. When considering the fold increase in drug solubility compared to the buffer as a function of the total amphiphile concentration in the simulated intestinal fluids, we observed a linear correlation for both indomethacin and felodipine (R^2^ = 0.97 and 0.92, respectively) (Figure 2B). Additionally, it is evident that the solubility of felodipine in Max‐PC was improved ≈10 times more than for indomethacin. Interestingly, a significantly higher apparent solubility was observed for felodipine in Max‐PC compared to Max‐lysoPC (104.7 ± 2.9 μg mL^−1^ and 78.5 ± 6.4 μg mL^−1^, respectively). This suggests that the type of lipid, and not only the total concentration, affects the solubility of more neutral and lipophilic drugs, such as felodipine, which makes this factor important to consider when investigating new formulations for oral drug delivery.

**Table 1:** Physicochemical characteristics for indomethacin and felodipine.^[^ ^27^ ^]^

Drug	M_W_ [g mol^−1^]	pKa	Charge (pH 6.5)	LogP
Indomethacin	357.8	4.42 (acid)	−1	3.51
Felodipine	384.3	NA	0	5.58

To further investigate the interactions between drugs and the simulated intestinal fluids, we compared the DLS size distribution of the prepared simulated intestinal fluids with and without the two drugs. Interestingly, we observed a clear grouping of the size distributions based on the observed solubilities (Figure 2C). The simulated intestinal fluids resulting in low drug solubility also resulted in either new peaks appearing or shifts in size distribution. On the other hand, we observed no changes in size, intensity, or distribution for the fluids, resulting in a high solubility. Additionally, the simulated intestinal fluids resulting in the largest fold solubility increase also consisted of more small colloidal structures (Figure 2C). This overall trend was confirmed when considering the relation between solubility and particle size (Figure 2D). Here, we observed a linear trend for indomethacin, but an exponential relation for felodipine. This highlights the complex nature of interactions between drug and colloidal structures in intestinal fluids, which depends on both drug‐specific characteristics (such as pKa and LogP value), but also on several environmental factors, including pH and levels of bile salts and lipids.

### 2.2 Particle Synthesis and Simulated Intestinal Fluid Interaction

To better understand how simulated intestinal fluids influence drug delivery systems, we investigated their interactions with nanoparticles exhibiting different properties. Specifically, we selected negatively charged polymersomes and mesoporous silica nanoparticles (MeSiNPs), including both negatively charged (n‐MeSiNPs) and positively charged (p‐MeSiNPs) variants. Polymersomes and MeSiNPs were chosen due to their versatility in surface chemistry modification, which allowed us to systematically study how different surface properties influence interactions with intestinal fluids, cells, and mucin. Polymersomes made from the copolymer PEG_22_‐p(CL_35_‐g‐TMC_35_) were synthesized via ring‐opening polymerization, with MSA serving as the catalyst. Polymersome formation was then triggered using the direct hydration method in phosphate‐buffered saline (PBS) (pH 6.5) at a concentration of 2 mg mL^−1^. MeSiNPs were synthesized using a co‐condensation approach, where a surfactant‐templated sol‐gel process was employed to control porosity and particle size.^[^ ^44^ ^]^ The synthesis involved the alkaline hydrolysis of a silica precursor, leading to the formation of n‐MeSiNP. To obtain p‐MeSiNP, surface functionalization was achieved through the addition of amine‐containing silanes, modifying the surface charge while maintaining the mesoporous structure.^[^ ^45^ ^]^ The surfactant template CTAB was removed by refluxing the nanoparticles in a mixture of HCl and ethanol.

**Figure 3:**
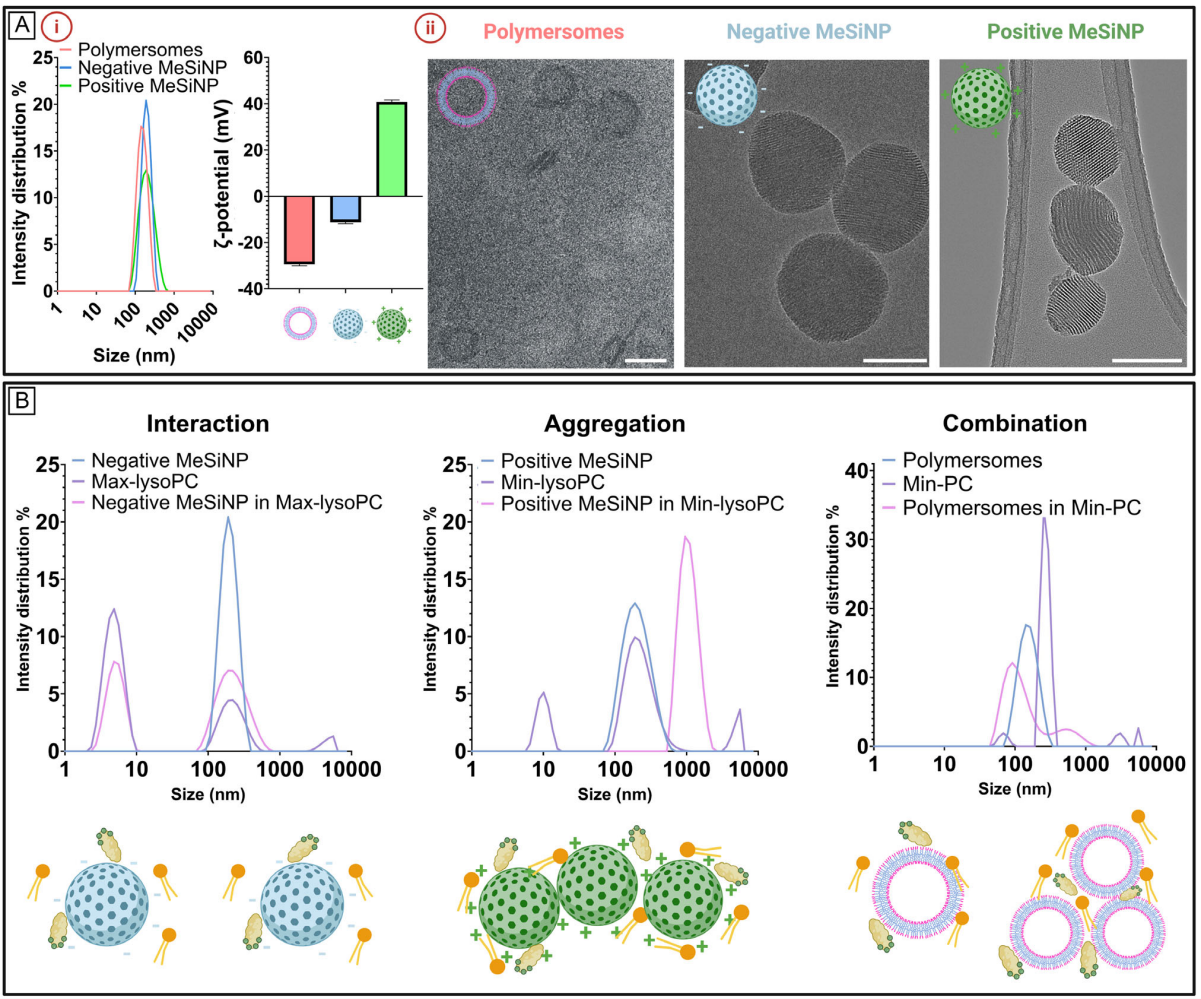
A) (i) Hydrodynamic size distribution and *ζ*‐potential measurements of the nanoparticles. (ii) Representative TEM images of the nanoparticles (scale bar: 100 nm). B) Representative examples illustrating distinct interaction behaviors between nanoparticles and simulated intestinal fluids.

All particles were resuspended at a concentration of 0.2 mg mL^−1^, and DLS analysis was performed. The reported sizes correspond to the intensity size of the main peak, with values of 173 ± 115.6 nm for the polymersomes, 233.5 ± 86.4 nm for n‐MeSiNP, and 215.6 ± 93.3 nm for p‐MeSiNP (**Figure** **3**A). The polydispersity indices (PDIs) were 0.201, 0.269, and 0.168, respectively. Similarly, the ζ‐potentials of the different particles were measured, yielding values of −29.43 ± 0.49 mV for polymersomes, −11.23 ± 0.65 mV for n‐MeSiNP, and 40.80 ± 0.65 mV for p‐MeSiNP (Figure 3A). These results suggest successful conjugation of APTES to the MeSiNP, leading to a positively charged surface for the p‐MeSiNP. To confirm the morphology of the nanoparticles, transmission electron microscopy (TEM) was used for MeSiNP and Cryo‐TEM for polymersomes. The images clearly showed the formation of vesicles for the polymersomes and confirmed the successful synthesis of both n‐MeSiNP and p‐MeSiNP with a pore size of 3 nm, which extended continuously across the full diameter of the nanoparticles (Figure 3A).

We then investigated the interaction between the particles and various simulated intestinal fluids to determine if these interactions could induce aggregation or size changes due to the particles’ interactions with constituents comprising the different fluids. This is particularly interesting because such changes could impact the stability of the nanoparticles, bioavailability, and overall effectiveness in drug delivery applications.^[^ ^46^, ^47^ ^]^ Additionally, the effects could vary from particle to particle, depending on the surface chemistry of each type, influencing their behavior and performance in physiological‐like conditions.^[^ ^12^, ^48^ ^]^

We incubated the particles in different simulated intestinal fluids for 30 min and analyzed the intensity distribution using DLS (Figure 3B). Based on the observations, we classified the interactions into distinct behaviors (**Table** **2**). The first, which we refer to as “interaction (I)”, was characterized by an increase in distribution around the main peak of the original particle size. While the average size remained largely unchanged, the peak broadened, indicating a shift in particle distribution. This suggests that components of the simulated intestinal fluids interact with the nanoparticle surface, potentially forming a biomolecular corona that could alter the particle's properties (Figure 3B).^[^ ^49^ ^]^ The second behavior, referred to as “aggregation (A)”, is characterized by a noticeable shift in the size distribution after incubation. This indicates that bile salts and lipids are facilitating the interaction and aggregation of the particles (Figure 3B). The final behavior, referred to as “combination (C)”, was identified when both interaction and aggregation occurred simultaneously within the same sample (Figure 3B).

**Table 2:** Simulated intestinal fluid–particle interactions classified as interaction (I), aggregation (a), or combination (C).

Nanoparticles	FaSSIF	Min‐PC	Max‐PC	Min‐lysoPC	Max‐lysoPC
Polymersomes	I	C	I	I	C
n‐MeSiNP	I/C	A	I	I	I
*p*‐MeSiNP	I	A	I	A	C

When comparing the results across the different simulated intestinal fluids and particles, we observed no consistent behavior that could be attributed to any single medium or particle type (other than Max‐PC, where we observed interaction for all types of particles). This suggests that the different components and concentrations in each simulated intestinal fluid can have an effect on the surface chemistry of the nanoparticles, thereby influencing their behavior. Therefore, the interactions between the particles and the components of the fluids are complex and depend not only on the inherent properties of the particles but also on the specific biochemical environment, which can influence the formation of biomolecular coronas, aggregation, or other particle‐mediated phenomena.

### 2.3 Cytotoxicity of Simulated Intestinal Fluids and Nanoparticles

**Figure 4:**
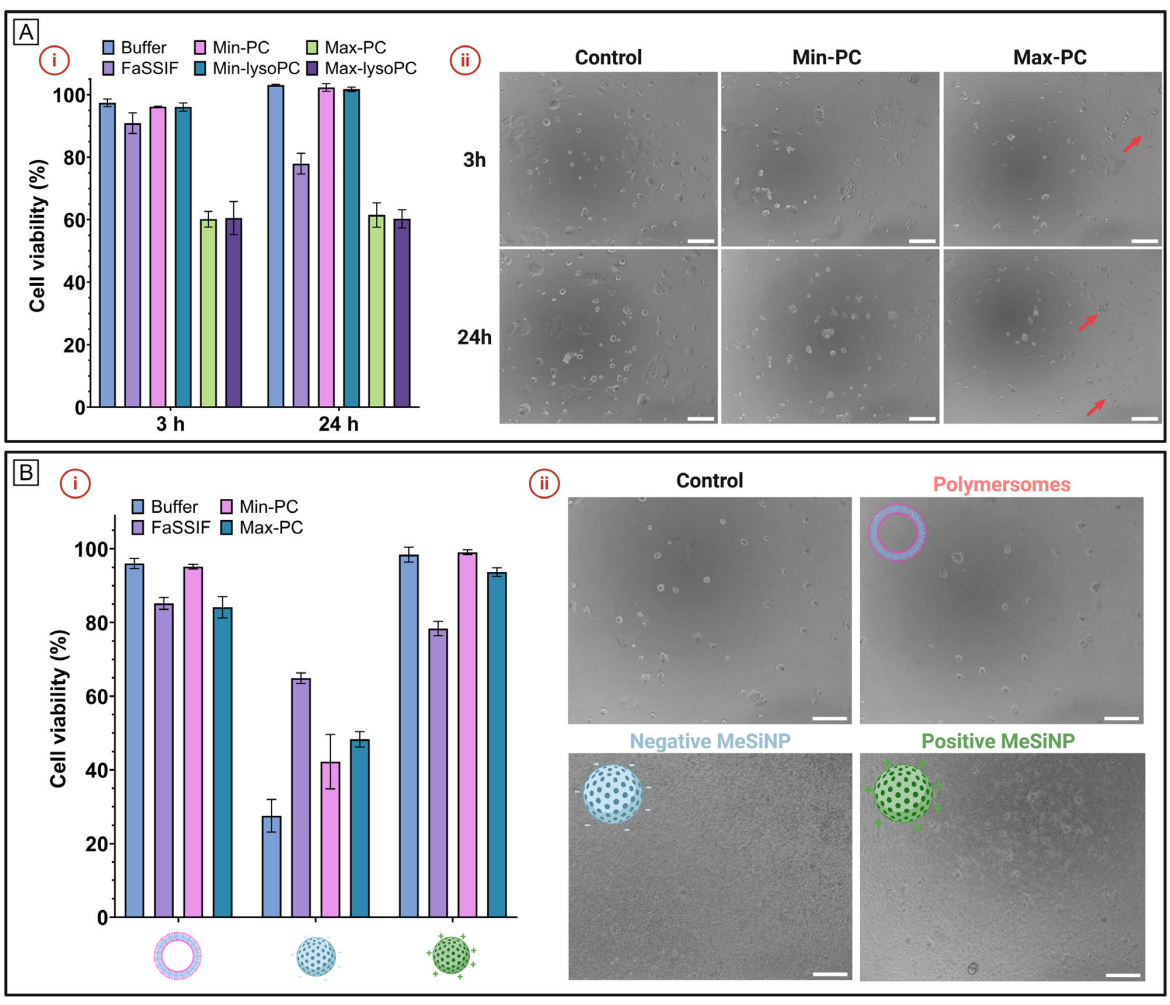
Cytotoxicity of the simulated intestinal fluids and nanoparticles was evaluated using Caco‐2 cells by LDH viability assay. A) (i) Relative cytotoxicity (%) of the buffer and the five simulated intestinal fluids, compared to a control in HBSS, after 3 and 24 h exposure, and (ii) Representative bright field images (10X) of Caco‐2 cells as a control and after exposure to Min‐PC and Max‐PC for 3 and 24 h, respectively. Red arrows highlight morphological signs of cytotoxicity, such as detachment of dead cells. B) (i) Relative cytotoxicity (%) of the three nanoparticles (polymersomes, negative MeSiNPs and positive MeSiNPS) in the buffer and selected simulated intestinal fluids after 3 h exposure, compared to a control in HBSS and (ii) Representative bright field images (10X) of Caco‐2 cells as control and Caco‐2 cells and nanoparticles in Min‐PC after 3 h. The error bars show ±SD (*n* = 4). All scale bars represent 100 μm.

We investigated how different simulated intestinal fluid compositions influence cytotoxicity in Caco‐2 cells, focusing on the impact of bile salts and lipid formulations. This aspect is relevant to consider, since the surface of nanoparticles is well‐known to affect how nanoparticles interact with cells.^[^ ^50^, ^51^ ^]^ Thus, the changes in surface interactions of the nanoparticles in different simulated intestinal fluids could also be expected to affect cellular interactions. As a general trend, we observed that higher bile salt concentrations correlated with increased toxicity, consistent with previous findings, where Max‐PC and Max‐lysoPC showed the highest toxicity, followed by FaSSIF (**Figure** **4**A). This toxicity was visually observed as both a reduced number of cells and changes in cell morphology, such as ballooning and detachment of dead cells (Figure 4A). Previously, similar cell viability percentages (≈70%) have been reported for Caco‐2 cells exposed to FaSSIF during 2 h,^[^ ^52^ ^]^ whereas for monolayers, commonly applied for drug permeability testing, no significant drop in cell viability was observed (up to 5 h).^[^ ^53^ ^]^ In contrast, no significant difference in toxicity was observed between Min‐PC and Min‐lysoPC or Max‐PC and Max‐lysoPC, suggesting that the type of lipid does not contribute substantially to cytotoxicity in this system. Based on these observations, we proceeded with the buffer and the simulated intestinal fluids exhibiting lower toxicity (FaSSIF, Min‐PC, and Min‐lysoPC), and incubated nanoparticles at 0.5 mg mL^−1^ to assess whether interactions or aggregation in different fluids influenced their cytotoxicity (Figure 4B). From the bright field images of particles incubated with Caco‐2 cells, we clearly observed the different nature of polymersomes and MeSiNPs (Figure 4B). However, these obvious morphological differences did not affect cell toxicity, as the polymersomes and p‐MeSiNPs showed no toxicity, while n‐MeSiNPs exhibited strong cytotoxic effects on Caco‐2 cells, which agrees with previous studies.^[^ ^54^, ^55^, ^56^ ^]^ Notably, polymersomes displayed increased toxicity in FaSSIF and Min‐lysoPC, but only in Min‐lysoPC did this exceed the toxicity of the simulated intestinal fluid alone, suggesting an additive effect due to interactions with the medium. A similar trend was observed for p‐MeSiNPs in FaSSIF, where incubation in the medium increased cytotoxicity. In contrast, n‐MeSiNPs demonstrated reduced toxicity in all simulated intestinal fluids compared to the PBS buffer, an unexpected finding given previous trends where interaction typically led to increased cytotoxicity. To interpret these findings, we compared cytotoxicity data with DLS results on nanoparticle behavior in buffer. In cases where interactions were observed via DLS, such as polymersomes in FaSSIF and Min‐lysoPC or p‐MeSiNPs in FaSSIF, cytotoxicity increased, likely due to surface chemistry modifications mediated by bile salts. However, when aggregation was predominant, such as with polymersomes in Min‐lysoPC or p‐MeSiNPs in Min‐PC and Min‐lysoPC, toxicity remained unchanged, suggesting that increased particle size alone does not drive cytotoxicity.^[^ ^57^ ^]^ For n‐MeSiNPs, the observed reduction in toxicity when resuspended in FaSSIF suggests that surface interactions can attenuate cytotoxic effects, while aggregation in other simulated intestinal fluids had less impact. These results highlight that surface chemistry alterations play a more significant role in cytotoxicity than nanoparticle size changes in colloidal systems.

### 2.4 Effect of Simulated Intestinal Fluids on Particles’ Interaction with Mucin

**Figure 5:**
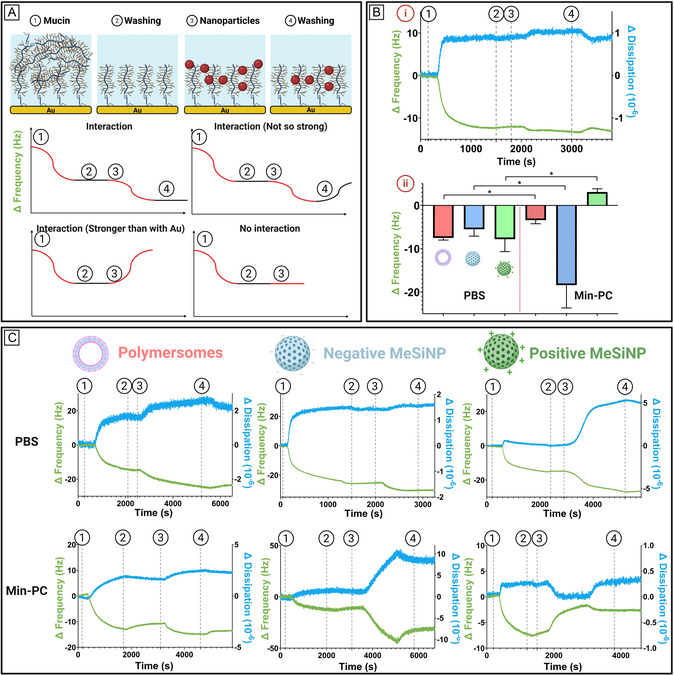
A) Schematic illustration of the four‐step procedure used to evaluate nanoparticle–mucin interactions and the possible interaction outcomes. B) (i) Representative QCM‐D data showing the interaction between Min‐PC and a mucin layer. (ii) Average frequency shift for nanoparticle–mucin interactions in PBS and Min‐PC (*N* = 3). C) Representative QCM‐D traces depicting interactions between various nanoparticles and a mucin‐coated gold sensor in PBS and Min‐PC. Frequency (green) and dissipation (blue) changes over time are shown for the fifth overtone (*N* = 3).

We then investigated how simulated intestinal fluid composition influences nanoparticle interactions with mucin using Quartz crystal microbalance with dissipation (QCM‐D). This surface‐sensitive technique allows real‐time quantification of changes in mass and viscoelastic properties on a sensor surface, making it particularly suitable for studying the strength of NPs binding to a mucin layer.^[^ ^58^ ^]^ We anticipated four possible outcomes (**Figure** **5**A) based on the interaction strength between the particles and mucin. The experiment followed four consecutive steps: first, mucin was adsorbed onto a gold sensor; second, a washing step removed loosely bound mucin; third, nanoparticles were introduced; and finally, another washing step was performed to remove loosely bound particles. The four expected interaction behaviors were: 1) strong interaction, where particles adhere firmly to the mucin layer and remain after the final wash; 2) weak interaction, where particles initially bind but are washed away; 3) mucin displacement, where the interaction between nanoparticles and mucin is stronger than that between mucin and the sensor, leading to partial mucin removal (since mucin is only adsorbed, not covalently bound, to the sensor); and 4) no interaction, where particles loosely associate with the mucin layer without significant retention (Figure 5A).^[^ ^59^, ^60^, ^61^ ^]^ To prevent pH‐induced conformational changes in mucin that could confound the results, all solutions were prepared with a pH of 6.5. Based on our cytotoxicity studies, we selected Min‐PC, which exhibited the lowest toxicity, for further investigation. First, we examined whether Min‐PC itself interacted with mucin, but no significant frequency changes were observed, indicating minimal to no interaction (Figure 5B). We then assessed nanoparticle interactions in both PBS and Min‐PC and observed distinct differences (Figure 5B). Interestingly, frequency shifts in PBS were relatively similar across all particle types, ranging from −8 to −12 Hz. However, when nanoparticles were incubated in Min‐PC, their behavior differed not only from their interactions in PBS but also from each other, indicating that intestinal fluid colloids influence not only particle behavior in solution and cell interactions but also their interactions with mucin. For polymersomes, we observed a reduction in frequency change, suggesting a weaker interaction with the mucin layer (Figure 5C). In contrast, both n‐MeSiNPs and p‐MeSiNPs exhibited stronger interactions, with p‐MeSiNPs even pulling mucin away from the sensor, indicating that their interaction with mucin surpassed mucin's adhesion to the gold sensor (Figure 5C).^[^ ^60^, ^61^ ^]^ Comparing these results to our DLS data, we found that nanoparticle aggregation correlated with stronger mucin interactions. For n‐MeSiNPs, this may result from larger aggregates accumulating on the sensor, increasing mass, and leading to a greater frequency shift.^[^ ^61^ ^]^ For p‐MeSiNPs, larger aggregates may be physically pulling mucin from the surface, causing a positive frequency shift. In contrast, polymersomes, which exhibited a mixed interaction pattern in DLS, displayed a weaker interaction with mucin. These findings suggest, in contrast to our cytotoxicity studies, that both surface chemistry modifications and colloidal size influence mucin interactions, highlighting the complexity of nanoparticle behavior in biological environments.

### 2.5 Effect of Simulated Intestinal Fluids on Polymer Interaction with Mucin

**Figure 6:**
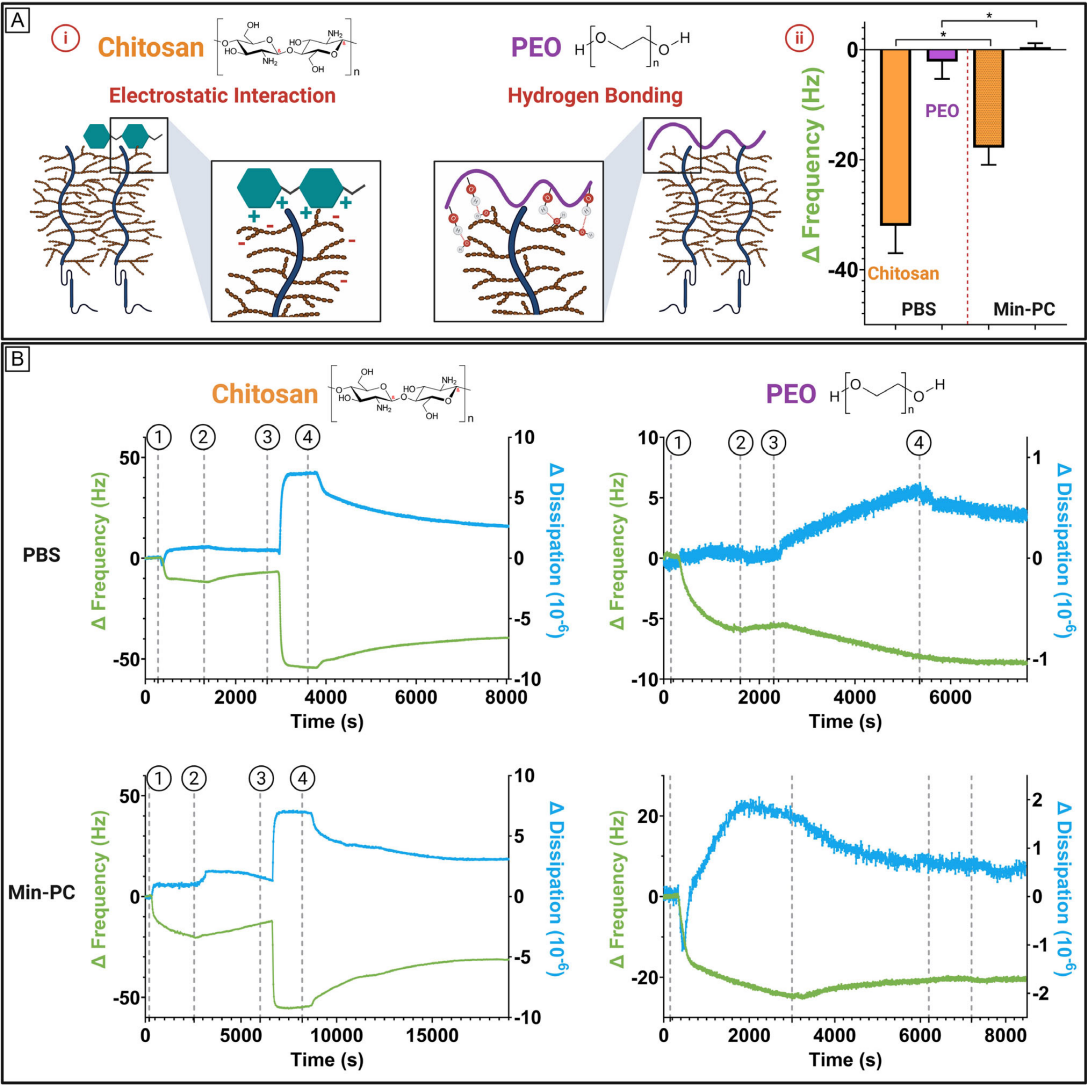
A (i) Schematic representation illustrating two distinct interaction modes between polymers and mucin. (ii) Average frequency shift observed for polymer–mucin interactions in PBS and Min‐PC (*N* = 3). B) Representative QCM ‐D traces showing the interaction of various polymers with a mucin‐coated gold sensor in PBS and Min‐PC. Frequency (green) and dissipation (blue) changes are shown over time for the fifth overtone (*N* = 3).

QCM‐D was further employed to investigate the interactions between polymers and mucin. In line with our previous experiments involving nanoparticles, we conducted similar measurements using chitosan and poly(ethylene oxide) (PEO) of similar molecular size. Both polymers are commonly used in oral drug delivery systems but interact with mucin through distinct mechanisms: chitosan primarily engages in electrostatic interactions due to its cationic nature, whereas PEO predominantly forms hydrogen bonds (**Figure** **6**A).^[^ ^62^, ^63^ ^]^ To some extent, the results indicated that both polymers exhibited mucoadhesive behavior in PBS (Figure 6B). However, their interactions with mucin changed markedly when incubated in Min‐PC. For chitosan, a notably smaller frequency shift was observed compared to the control in PBS, indicating a reduced interaction with mucin. A similar trend was observed with PEO, albeit to a greater extent, where no measurable frequency shift was detected upon its addition to Min‐PC, indicating no interaction with mucin. This suggests that PEO's interaction with mucin was effectively shielded, likely due to competing interactions between the polymer and components of Min‐PC, which prevented any interaction with mucin. (Figure 6B).

It is particularly interesting to observe how polymersome nanoparticles based on block copolymers that include poly(ethylene glycol) (PEG) interact with the system and PEO‐polymers that share the same chemical structure but differ in molecular weight and, in this case, colloidal organization, exhibit comparable mucoadhesive behavior when suspended in PBS. This suggests that despite differences in size and morphology, both systems can engage with mucin under physiological‐like salt conditions. However, when the medium is changed to Min‐PC, the interaction is significantly altered: where for the free polymer (PEO), no measurable interaction with mucin is observed, indicating that the simulated intestinal fluid components are likely to screen or compete with hydrogen bonding or other weak interactions, while the nanoparticle still shows some degree of interaction. This contrast is particularly insightful when considering PEG‐coated nanoparticles, which are widely described as mucopenetrating rather than mucoadhesive.^[^ ^64^, ^65^ ^]^ The findings suggest that PEG‐based nanoparticles may exhibit apparent mucoadhesion under specific conditions (e.g., in low‐ionic‐strength buffers like PBS) but lose these interactions in more physiologically relevant environments such as Min‐PC. This underscores the importance of buffer composition in mucoadhesion studies and highlights that interpreting PEG‐related interactions requires careful consideration of the surrounding media.^[^ ^12^ ^]^ Moreover, it raises important questions about how PEG coatings on nanoparticles behave in complex in vivo mucus environments, whether they truly prevent interactions with mucin or if such interactions are merely masked under specific conditions, potentially changing with varying GI environments.

## 3 Conclusion

To date, studies on biomolecular interactions have primarily focused on protein interactions in the plasma environment, whereas literature on interactions with intestinal biomolecules remains limited.^[^ ^12^, ^33^ ^]^ In this work, our objective was to study how different components involved in oral drug delivery, from the drug compound itself to polymers and nanoparticles, interact with the colloidal structures formed by bile salts and phospholipids in intestinal fluid. First, we studied the interactions between simulated intestinal fluids and two poorly water‐soluble drugs, indomethacin and felodipine. Both drugs showed increasing apparent solubility with higher total concentration of amphiphiles. Interestingly, felodipine revealed a significantly higher solubility in the presence of lysoPC compared to PC, revealing that the type of lipid applied in simulated intestinal fluids can affect solubility. When comparing solubility and DLS data, we observed that for the simulated intestinal fluids resulting in low drug solubility, new peaks appeared or there was a shift in size distribution compared to the pure simulated intestinal fluid. On the other hand, we observed no changes in size, intensity, or distribution for the fluids, resulting in a high solubility.

When we investigated interactions between the simulated intestinal fluids and polymersomes, n‐MeSiNPs and p‐MeSiNPs using DLS, we could classify the interactions into three categories based on shifts in size distribution: 1) interaction, 2) aggregation, and 3) combination. This could not be grouped or attributed to any single medium or particle type. When the nanoparticles were incubated with Caco‐2 cells in the different simulated intestinal fluids, we observed an interesting relation between the DLS categories and cytotoxicity. In cases where “interaction” was observed via DLS, the cytotoxicity increased, and when aggregation was predominant, cytotoxicity remained unchanged.

We further investigated nanoparticle‐media synergies using QCM‐D to study interactions with mucins in the presence and absence of simulated intestinal fluids. Interestingly, frequency shifts in PBS were relatively similar across all particle types, but when the nanoparticles were incubated in simulated intestinal fluids, their behavior differed not only from their interactions in PBS but also from each other. When we explored interactions between chitosan and PEO with mucin, we observed mucoadhesive behavior for both polymers in PBS. However, in the presence of simulated intestinal fluid, both chitosan and PEO showed a reduction in their interactions with mucin. While chitosan retained some of its mucoadhesive properties, the most significant change was seen with PEO, where no interaction with mucin was observed.

All in all, these results stress the importance of screening, not only drug properties, but also nanoparticles and polymers, in the presence of intestinal colloidal structures. The interactions are complex and will depend on both particle/polymer surface chemistry and changes in colloidal size, as well as the specific biochemical environment in (simulated) intestinal fluids. Ideally, future studies should incorporate a broader range of nanoparticles, varying not only in material composition but also in shape, size, and ligand density, as these parameters are known to influence mucosal diffusion.^[^ ^66^, ^67^ ^]^ In addition, merging studies on drug encapsulation with nanoparticle transport behavior, particularly considering how the localization of hydrophobic or hydrophilic drugs within the nanocarrier can modulate mucin interactions, will be essential for building a more comprehensive understanding of nanoparticle, mucus interplay under physiologically relevant conditions. Further, transitioning toward the use of human GI aspirates, comparing both fasted and fed conditions, would further enhance physiological relevance.

## 4 Experimental Section

4.1

4.1.1

##### Materials

All chemicals were used as received unless otherwise stated. Monomethoxy‐poly(ethylene glycol), 1 kDa (≥95%), was obtained from JenKem Technology USA. Monomer trimethylene carbonate was purchased from TCI Europe (>98%). Monomer ε‐caprolactone (99%) was purchased from Fluorochem. Taurocholic acid sodium salt hydrate was acquired from ACROS Organics (now Thermo Fisher Scientific, Waltham, MA, USA). 3 F powder (FF01) was purchased from Biorelevant.com (London, UK). PC (purity: 99.1%) and lysoPC (purity: 80%), both from soybean oil, were kindly donated by Lipoid (Ludwigshafen, Germany). All other chemicals and reagents, including cetrimonium bromide (CTAB), (3‐aminopropyl)triethoxysilane (APTES), tetraethyl orthosilicate (TEOS), methanesulfonic acid (MSA) (≥99%), triethylamine (≥99.5%), chitosan (190 000–310 000 Da), PEO (200 kDa), mucin from porcine stomach Type III (PGM), PEG350, PBS, dichloromethane (DCM), toluene, chloroform, acetic acid, sodium carbonate (NaHCO_3_) sodium sulfate (Na_2_SO_4_), KOH, sodium chloride and sodium dihydrogen monophosphate dihydrate were purchased from Sigma–Aldrich (St. Louis, MO). Unless otherwise indicated, all cell culture reagents were acquired from Thermo Fisher Scientific (Waltham, MA, USA). Solvents were HPLC grade and used as received. Ultrapure water was used throughout the experiments and obtained from Merck Millipore Q‐Pod system (Merck Group, Burlington, MA, USA) with a 0.22 μm Millipore Express 40 filter (18.2 MΩ).

##### Cell Line and Subculturing

The human colorectal adenocarcinoma cell line Caco‐2 (HTB‐37) was purchased from the American Type Culture Collection (ATCC, Manassas, VA, USA). The Caco‐2 cells were cultured in Dulbecco's modified Eagle medium containing glucose (4.5 g L^−1^) supplemented with heat‐inactivated fetal bovine serum (10% v/v), nonessential amino acids (1 % v/v), streptomycin (100 μg/mL), and penicillin (100 U mL^−1^). The cells were maintained in a humidified incubator at 37 °C with 5% CO_2_. The cells were subcultured once a week using trypsin/ethylenediamine tetraacetic acid (0.05%) and counted using an automated cell counting unit (NucleoCounter NC‐200TM, ChemoMetec A/S, Allerod, Denmark). Cells from passages 12–15 were used for experiments.

##### Data and Statistical Analysis

Data analysis was performed with GraphPad Prism (Version 9.4.1 (681) Insight Partners, Graphpad Holdings, LLC, New York City, NY, USA). Data were presented as average and standard deviation (SD) where *n* represents the number of repetitions within each sample and *N* represents the number of samples. Statistical analysis was performed for statistically significant differences (*p < 0.05) with GraphPad Prism using a t‐test (for two independent populations) or one‐way analysis of variance (ANOVA) (three or more independent populations). The figures were created with Biorender.com. Image analysis and processing were done using the software ImageJ (version 1.53t, National Institutes of Health, USA).

##### Preparation and Characterization of Simulated Intestinal Fluids

The simulated intestinal fluids were prepared following a previously published protocol^[^ ^40^ ^]^ to achieve the desired compositions (**Table** **3**). Briefly, the required amount of PC or lysoPC stock in chloroform (30 mM) was pipetted, and a lipid film was formed by complete evaporation of the solvent. Sodium taurocholate was added to achieve the desired bile salt concentrations. The buffer was prepared from sodium dihydrogen monophosphate (24.9 mm) and sodium chloride (52.9 mm) to achieve the desired buffer capacity and osmolarity. The commercial FaSSIF was prepared according to the manufacturer's instructions (Biorelevant.com, London, UK) with the same buffer as the other fluids. The simulated intestinal fluids were stirred overnight at 37 °C, and the next day, the pH was adjusted to pH 6.5. The hydrodynamic colloidal size distribution of the buffers was determined using a Malvern Zetasizer Nano ZSP (Malvern Instruments, Worcestershire, UK) with single‐use disposable microcuvettes. Measurements were conducted without dilution or filtration. DLS analysis was performed using 100 μL of the sample, a detection angle of 173°, and 13 runs of 10 s each (*N *= 3).

**Table 3:** Overview of bile salt (BS) and phospholipid (PL) concentration in the five prepared simulated intestinal fluids.

	Sodium taurocholate [mm]	PC [mm]	LysoPC [mm]	BS/PL
FaSSIF	3.0	0.75	–	4.0
Min‐PC	0.4	0.03	–	13.3
Min‐lysoPC	0.4	–	0.03	13.3
Max‐PC	10.0	1.50	–	6.7
Max‐lysoPC	10.0	–	1.50	6.7

##### Characterization of Drugs–Dissolution, Solubility and Size

The dissolution and apparent solubilities of two poorly water‐soluble drugs, indomethacin and felodipine, were determined in the simulated intestinal fluids using a MicroDISS Profiler (pION Inc Ltd, East Sussex, UK). In each experiment (*n* ≥ 3), excess powder (at least 2‐3 times) compared to the expected solubility value was added to 10 mL simulated intestinal fluid. The experiments were performed at 37 °C using a stirring rate of 150 rpm, while the samples were scanned by individual UV probes every 10–30 s. Depending on the expected solubility of each compound in the different simulated intestinal fluids, the path length of the in situ UV probe varied between 1 and 5 mm. Prior to the experiment, each individual channel was calibrated with a separate calibration curve by the addition of aliquots of a dimethyl sulfoxide stock solution of the relevant drug compound to 10 mL simulated intestinal fluid. The concentrations were determined based on the area under the curve in second derivative spectra evaluated over a range of wavelengths. This method minimizes potential interference from background turbidity caused by the powder.^[^ ^27^ ^]^ The hydrodynamic size of the simulated intestinal fluids and drugs dissolved in the different simulated intestinal fluids was determined using a Malvern Zetasizer Nano ZSP (Malvern Instruments, Worcestershire, UK) with single‐use disposable microcuvettes. Measurements were conducted without dilution or filtration. DLS analysis was performed using 100 μL of the sample, a detection angle of 173°, and 13 runs of 10 s each (*N *= 3).

##### Nanoparticle Synthesis

Silanol (–SiOH) and amine (–NH_2_) functionalized MeSiNPs were synthesized using a modified co‐condensation method, yielding negatively and positively charged nanoparticles, respectively.^[^ ^44^ ^]^ CTAB (0.25 g, 0.69 × 10^−3^ mol) was first dissolved in 120 mL of MilliQ water. Subsequently, 0.75 mL of NaOH (2 m) was added to the solution, which was stirred for 5 min before adjusting the temperature to 80 °C. TEOS (1.25 mL, 5.6 × 10^−3^ mol) was then added dropwise to the surfactant solution. For amine (–NH_2_) functionalized MeSiNPs, APTES (0.25 mL, 1.07 × 10^−3^ mol) was introduced dropwise. The mixture was stirred for 2 h, resulting in the formation of white precipitates. The solid product was collected by centrifugation, washed with deionized water and ethanol, and then dried under a nitrogen stream. To remove the CTAB surfactant template, 0.5 g of MeSiNPs were refluxed for 24 h in a solution of 3 mL HCl (34%) and 50 mL ethanol. The particles were subsequently washed extensively with deionized water and ethanol and dried under nitrogen for 24 h. The samples were stored at 4 °C in Eppendorf tubes wrapped in aluminum foil to protect them from light. For the polymersomes, the block copolymer PEG_22_‐p(CL_38_‐g‐TMC_37_) was synthesized following a previously published procedure.^[^ ^54^, ^68^ ^]^ Monomethoxy‐poly(ethylene glycol) (*M*_*n*_ ≈ 1000 g mol^−1^; 705.2 mg, 0.71 mmol), ε‐caprolactone (εCL, 2.97 mL, 26.8 mmol), and trimethylene carbonate (TMC, 2.66 g, 26.1 mmol) were dissolved in anhydrous toluene, concentrated under vacuum twice to remove water, and redissolved in dry DCM (150 mL). MSA (0.1 eq. to εCL) was added, and the mixture was stirred at room temperature for 24 h. After reaction, the solution was washed with saturated NaHCO_3_, water, and brine, dried over Na_2_SO_4_, filtered, and concentrated under vacuum. The product was an oily, colorless material, which was lyophilized from 1,4‐dioxane for 2 days to yield a waxy oil.

The polymer stock solution, PEG_22_‐p(CL_38_‐g‐TMC_37_), was dissolved in PEG350 at 1% (w/w) in a heated bath for 30 min. A 20 μL volume of this solution was added to a glass vial, and 80 μL of PBS (pH 6.5) was rapidly mixed in. The solution became opaque, and after 5 min of stirring, PBS was gradually added to reach the desired concentration. The samples were stored at 4 °C in Eppendorf tubes (2 mg mL^−1^) wrapped in aluminum foil to protect them from light.

##### Characterization of Nanoparticles–Size, Surface Charge and Morphology

The hydrodynamic size, PDI, and ζ‐potential of the nanoparticles were measured using a Malvern Zetasizer Nano ZS (Malvern Instruments, Worcestershire, UK) in single‐use disposable microcuvettes to confirm successful synthesis and assembly. Measurements were performed in PBS adjusted to pH 6.5 at a nanoparticle concentration of 0.2 mg mL^−1^. DLS was conducted on 100 μL of the sample, a 173° detection angle, 13 runs of 10 s per run, and three measurements. The ζ‐potential was assessed by laser Doppler electrophoresis using the Zetasizer Nano ZS, with 700 μL of the sample in folded capillary cells and 10 runs over three measurements (*N* = 3, *n *= 3). The size and morphology of the nanoparticles were confirmed using TEM on a FEI Tecnai G2 T20 (FEI Company, Hillsboro, OR, USA) operating at 200 keV in low‐dose mode with a TVIPS XF416 CCD camera. For MeSiNPs, TEM analysis was used, where 3 μL of the NP solution was pipetted onto lacey carbon‐coated 300 mesh copper grids. To confirm size and morphology for the polymersomes, cryo‐TEM samples were prepared by glow‐discharging Quantifoil Cu grids with holey carbon films in a Cressington 208 carbon coater for 40 s. After adding 3 μL of the nanoparticle solution, the grid was blotted in a Vitrobot MARK IV (FEI Company) at room temperature and 100% humidity, then vitrified in liquid ethane. Images were captured with zero‐loss energy filtering (Gatan GIF 2002, 20 eV energy slit) on a CCD camera (Gatan model 794). Image analysis was performed using ImageJ software, with NP width determined by the “line” function followed by the “measure” option. Size is reported as average ± SD (*N *> 15).

##### Simulated Intestinal Fluid–Particle Interaction

The hydrodynamic size of the nanoparticles in various buffers was measured using a Malvern Zetasizer. The simulated intestinal fluids were prepared as previously described, and the three different types of nanoparticles were suspended in each buffer at a final concentration of 0.2 mg mL^−1^, then incubated for 30 min before measurements. DLS was conducted with 100 μL of the sample, a 173° detection angle, 13 runs of 10 s per run, and three measurements.

##### Cytotoxicity Studies

The cell viability was assessed using a lactate dehydrogenase (LDH) based cytotoxicity kit (LDH Cytotoxicity Detection Kit^PLUS^, Roche, Basel, Switzerland) according to the manufacturer's protocol. Caco‐2 cells were seeded in a 96‐well plate at a density of 5,000 cells/well. After 24 h culture, the culture medium was exchanged with the five different simulated intestinal fluids at time points resulting in 3 and 24 h exposure time to evaluate the toxicity of the buffer and the five simulated intestinal fluids. At the same time points, Hank's Balanced Salt Solution (HBSS) was added to the control wells. To evaluate the cell viability after exposure to the nanoparticles in different simulated intestinal fluids, the particles were pre‐suspended in the fluids at 0.5 mg mL^−1^ before adding the suspension to the cells for 3 h. The absorbance was measured at 490 nm (background wavelength 680 nm) using a multimode microplate reader (Thermo Fisher Scientific, Waltham, MA, USA). The relative cell viability (%) was expressed as a ratio to control groups (*N *= 4).

##### QCM‐D Studies

QCM‐D measurements were performed using a Q‐Sense E4 system (Gothenburg, Sweden) equipped with gold‐coated quartz crystals (QSX301, Q‐Sense, Gothenburg, Sweden) with a fundamental frequency of 4.95 Hz.^[^ ^69^, ^70^ ^]^ Before each experiment, the sensors were cleaned by sequentially rinsing with sodium dodecyl sulfate (2% w/v), ultrapure water, and ethanol, followed by drying with N_2_ gas. Experiments were carried out in flow mode at 37.0 °C, maintaining a flow rate of 50 μL min^−1^. The system simultaneously recorded changes in frequency (Δ*f*) and energy dissipation factor (Δ*D*) at the 3rd, 5th, 7th, 9th, 11th, and 13th overtones. All measurements were conducted at pH 6.5. For the control experiments, mucin and nanoparticles were prepared in PBS at concentrations of 0.3 mg mL^−1^ and 0.2 mg mL^−1^, respectively. To initiate the experiment, PBS was introduced to stabilize the Δ*f* and Δ*D* signals. Mucin was then flowed into the system, allowing its adsorption onto the sensor surface, followed by a PBS rinse to remove unbound mucin. Once the signal stabilized, nanoparticles were introduced to assess their interaction with the mucin layer, and the flow was maintained until a steady state was reached. Finally, a PBS wash was performed to remove any unbound nanoparticles, ensuring signal stabilization.

For experiments conducted in simulated intestinal fluids, the same protocol was applied, with nanoparticles suspended at 0.2 mg mL^−1^ in simulated intestinal fluid instead of PBS. Additionally, to examine the interaction between the simulated intestinal fluids and mucin, an identical procedure was followed, except that nanoparticles were replaced with blank simulated intestinal fluid addition. All other conditions remained unchanged. The data presented in this study include a representative QCM‐D graph for the fifth harmonic overtone, along with the average change in Δ*f* (*N* = 3). A similar procedure was followed for the studies with chitosan and PEO, which were dissolved at a concentration of 0.2 mg mL^−1^ in PBS or simulated intestinal fluid. The data presented in this study include a representative QCM‐D graph for the fifth harmonic overtone, along with the average change in Δ*f* (*N* = 3).

## Conflict of Interest

The authors declare no conflict of interest.

## Author Contributions

**Juliane Fjelrad Christfort**: conceptualization (equal); data curation (equal); formal analysis (equal); investigation (equal); methodology (equal); validation (equal); visualization (equal); writing—original draft (equal); writing—review and editing (equal). **Matteo Tollemeto**: conceptualization (equal); data curation (equal); formal analysis (equal); investigation (equal); methodology (equal); validation (equal); visualization (equal); writing—original draft (equal); writing—review and editing (equal). **Yudong Li**: investigation (supporting); methodology (supporting). **Lasse Højlund Eklund Thamdrup**: supervision (supporting); writing—review and editing (supporting). **Jan van Hest**: supervision (supporting); writing—review and editing (supporting). **Anja Boisen**: funding acquisition (lead); project administration (lead); supervision (lead); writing—review and editing (supporting). **Juliane Fjelrad Christfort** and **Matteo Tollemeto** contributed equally to this work.

## Data Availability

The data that support the findings of this study are available from the corresponding author upon reasonable request.

## References

[1] C. Milián‐Guimerá , R. McCabe , L. H. E. Thamdrup , M. Ghavami , A. Boisen , J. Controlled Release 2023, 364, 227. 10.1016/j.jconrel.2023.10.041 39491170

[2] N. K. Mandsberg , J. F. Christfort , K. Kamguyan , A. Boisen , S. K. Srivastava , Adv. Drug Deliv. Rev. 2020, 165, 142. 32416112 10.1016/j.addr.2020.05.004 PMC7255201

[3] M. J. Mitchell , M. M. Billingsley , R. M. Haley , M. E. Wechsler , N. A. Peppas , R. Langer , Nat. Rev. Drug Discovery 2020 20:2 2020, 20, 101. 33277608 10.1038/s41573-020-0090-8 PMC7717100

[4] R. Awasthi , G. T. Kulkarni , Drug Deliv. 2016, 23, 378. 25026414 10.3109/10717544.2014.936535

[5] J. Tripathi , P. Thapa , R. Maharjan , S. H. Jeong , Pharmaceutics 2019, 11, 193. 31010054 10.3390/pharmaceutics11040193 PMC6523542

[6] A. Banerjee , J. Qi , R. Gogoi , J. Wong , S. Mitragotri , J. Controlled Release 2016, 238, 176. 10.1016/j.jconrel.2016.07.051 PMC5289391 27480450

[7] N. Zhang , J. Song , Y. Han , Biomolecules 2024, 14, 1628. 39766335 10.3390/biom14121628 PMC11726895

[8] C. A. S. Bergström , R. Holm , S. A. Jørgensen , S. B. E. Andersson , P. Artursson , S. Beato , A. Borde , K. Box , M. Brewster , J. Dressman , K. Feng , G. Halbert , E. Kostewicz , M. McAllister , U. Muenster , J. Thinnes , R. Taylor A. Mullertz , Eur. J. Pharm. Sci. 2013, 57, 173. 24215735 10.1016/j.ejps.2013.10.015

[9] S. Hua , Front. Pharmacol. 2020, 11, 524. 32425781 10.3389/fphar.2020.00524 PMC7212533

[10] M. P. Moreno , J. Pharm. Pharmacol. 2010, 58, 1079.

[11] J. B. Dressman , R. R. Berardi , L. C. Dermentzoglou , T. L. Russell , S. P. Schmaltz , J. L. Barnett , K. M. Jarvenpaa , Pharm. Res. 1990, 7, 756. 2395805 10.1023/a:1015827908309

[12] A. Aljabbari , S. Kihara , T. Rades , B. J. Boyd , J. Controlled Release 2023, 363, 536. 10.1016/j.jconrel.2023.09.049 37776905

[13] A. Lindahl , A. L. Ungell , L. Knutson , H. Lennernäs , Pharm. Res. 1997, 14, 497. 9144738 10.1023/a:1012107801889

[14] H. P. Simonian , L. Vo , S. Doma , R. S. Fisher , H. P. Parkman , Dig. Dis. Sci. 2005, 50, 2276. 16416175 10.1007/s10620-005-3048-0

[15] S. Clarysse , J. Tack , F. Lammert , G. Duchateau , C. Reppas , P. Augustijns , J. Pharm. Sci. 2009, 98, 1177. 18680176 10.1002/jps.21502

[16] M. P. de la Cruz‐Moreno , C. Montejo , A. Aguilar‐Ros , W. Dewe , B. Beck , J. Stappaerts , P. Augustijns , J. Tack , Int. J. Pharm. 2017, 528, 471. 28591618 10.1016/j.ijpharm.2017.05.072

[17] G. Vitiello , D. Ciccarelli , O. Ortona , G. D’Errico , J. Colloid Interface Sci. 2009, 336, 827. 19414180 10.1016/j.jcis.2009.04.008

[18] M. C. Carey , D. M. Small , Md Boston , Am. J. Med. 1970, 49, 590. 4924587 10.1016/s0002-9343(70)80127-9

[19] D. Madenci , S. U. Egelhaaf , Curr. Opin. Colloid Interface Sci. 2010, 15, 109.

[20] A. F. Hofmann , L. R. Hagey , Cell. Mol. Life Sci. 2008, 65, 2461. 18488143 10.1007/s00018-008-7568-6 PMC11131813

[21] V. Forooqi Motlaq , M. Ortega‐Holmberg , K. Edwards , L. Gedda , J. Lyngsø , J. S. Pedersen , L. M. Bergström , Soft Matter 2021, 17, 7769. 34351343 10.1039/d1sm00745a

[22] P. A. Elvang , A. H. Hinna , J. Brouwers , B. Hens , P. Augustijns , M. Brandl , J. Pharm. Sci. 2016, 105, 2832. 27103012 10.1016/j.xphs.2016.03.005

[23] M. Leigh , B. Kloefer , M. Schaich , Dissolut. Technol. 2013, 20, 44.

[24] L. Klumpp , K. Nagasekar , O. Mccullough , A. Seybert , M. Ashtikar , J. Dressman , Dissolut. Technol. 2019, 26, 6.

[25] Z. Mckinnon , I. Khadra , G. W. Halbert , H. K. Batchelor , Int. J. Pharm. 2024, 665, 124733. 39317247 10.1016/j.ijpharm.2024.124733

[26] A. Teleki , O. Nylander , C. A. S. Bergström , Pharmaceutics 2020, 12, 493. 32481718 10.3390/pharmaceutics12060493 PMC7356998

[27] J. H. Fagerberg , O. Tsinman , N. Sun , K. Tsinman , A. Avdeef , C. A. S. Bergström , Mol. Pharm. 2010, 7, 1419. 20507160 10.1021/mp100049m

[28] S. Hanio , S. Möllmert , C. Möckel , S. Choudhury , A. I. Höpfel , T. Zorn , S. Endres , J. Schlauersbach , L. Scheller , C. Keßler , O. Scherf‐Clavel , P. Bellstedt , U. Schubert , A. Poppler , K. Heinze , J. Guck L. Meinel , Mol. Pharm. 2023, 20, 6151. 37906224 10.1021/acs.molpharmaceut.3c00550

[29] K. L. May , K. J. Tangso , A. Hawley , B. J. Boyd , A. J. Clulow , Food Hydrocoll. 2020, 108, 105965.

[30] S. Endres , E. Karaev , S. Hanio , J. Schlauersbach , C. Kraft , T. Rasmussen , R. Luxenhofer , B. Böttcher , L. Meinel , A. C. Pöppler , J. Colloid Interface Sci. 2022, 606, 1179. 34487937 10.1016/j.jcis.2021.08.040

[31] J. Schlauersbach , S. Hanio , B. Lenz , S. P. B. Vemulapalli , C. Griesinger , A. C. Pöppler , C. Harlacher , B. Galli , L. Meinel , J. Controlled Release 2021, 330, 36. 10.1016/j.jconrel.2020.12.016 33333120

[32] M. Lundqvist , T. Cedervall , M. Lundqvist , T. Cedervall , Small 2020, 16, 2000892. 10.1002/smll.202000892 33107223

[33] S. Kihara , A. Aljabbari , K. Bērziņš , L. S. Krog , P. Mota‐Santiago , A. Terry , N. Kirby , A. E. Whitten , B. J. Boyd , J. Colloid Interface Sci. 2025, 680, 797. 39591792 10.1016/j.jcis.2024.11.064

[34] D. Riethorst , R. Mols , G. Duchateau , J. Tack , J. Brouwers , P. Augustijns , J. Pharm. Sci. 2016, 105, 673. 26228456 10.1002/jps.24603

[35] K. Pyper , J. Brouwers , P. Augustijns , I. Khadra , C. Dunn , C. G. Wilson , G. W. Halbert , Eur. J. Pharm. Biopharm. 2020, 153, 226. 32585351 10.1016/j.ejpb.2020.06.011

[36] C. M. Madsen , J. Plum , B. Hens , P. Augustijns , A. Müllertz , T. Rades , J. Pharm. Sci. 2021, 110, 2479. 33428916 10.1016/j.xphs.2020.12.039

[37] A. Fuchs , J. B. Dressman , J. Pharm. Sci. 2014, 103, 3398. 25277073 10.1002/jps.24183

[38] D. Psachoulias , M. Vertzoni , K. Goumas , V. Kalioras , S. Beato , J. Butler , C. Reppas , Pharm. Res. 2011, 28, 3145. 21674262 10.1007/s11095-011-0506-6

[39] E. Persson , L. Löfgren , G. Hansson , B. Abrahamsson , H. Lennernäs , R. Nilsson , J. Lipid Res. 2007, 48, 242. 17062898 10.1194/jlr.D600035-JLR200

[40] J. F. Christfort , S. Strindberg , J. Plum , J. Hall‐Andersen , C. Janfelt , L. H. Nielsen , A. Müllertz , Eur. J. Pharm. Biopharm. 2019, 142, 307. 31288077 10.1016/j.ejpb.2019.07.007

[41] O. Pabois , R. M. Ziolek , C. D. Lorenz , S. Prévost , N. Mahmoudi , M. W. A. Skoda , R. J. L. Welbourn , M. Valero , R. D. Harvey , M. M. L. Grundy , P. Wilde , I. Grillo , Y. Gerelli C. Dreiss , J. Colloid Interface Sci. 2021, 587, 522. 33189321 10.1016/j.jcis.2020.10.101

[42] C. de Oliveira , B. Khatua , B. El‐Kurdi , K. Patel , V. Mishra , S. Navina , B. J. Grim , S. Gupta , M. Belohlavek , B. Cherry , J. Yarger , M. Green V. Singh , Sci. Rep. 2020 10:1 2020, 10, 10.1038/s41598-020-65451-w. PMC7242474 32439972

[43] M. U. Ahmad , S. M. Ali , A. Ahmad , S. Sheikh , I. Ahmad , Polar Lipids: Biology, Chem. Technol. 2015, 349–389, 10.1016/B978-1-63067-044-3.50015-7.

[44] S. L. Burkett , S. D. Sims , S. Mann , Chem. Commun. 1996, 11, 1367.

[45] C. Von Baeckmann , R. Guillet‐Nicolas , D. Renfer , H. Kählig , F. Kleitz , ACS Omega 2018, 3, 17496. 31458354 10.1021/acsomega.8b02784 PMC6644079

[46] W. Inam , R. Bhadane , R. N. Akpolat , R. A. Taiseer , S. K. Filippov , O. M. H. Salo‐Ahen , J. M. Rosenholm , H. Zhang , View 2022, 3, 20210009.

[47] M. Lundqvist , C. Cabaleiro‐Lago , J Colloid Interface Sci 2017, 504, 78. 28527828 10.1016/j.jcis.2017.05.031

[48] S. Li , C. Cortez‐Jugo , Y. Ju , F. Caruso , ACS Nano 2024, 18, 33257. 39602410 10.1021/acsnano.4c13214

[49] J. Stetefeld , S. A. McKenna , T. R. Patel , Biophys. Rev. 2016, 8, 409. 28510011 10.1007/s12551-016-0218-6 PMC5425802

[50] A. Verma , F. Stellacci , Small 2010, 6, 12. 19844908 10.1002/smll.200901158

[51] A. E. Nel , L. Mädler , D. Velegol , T. Xia , E. M. V. Hoek , P. Somasundaran , F. Klaessig , V. Castranova , M. Thompson , Nat. Mater. 2009 8:7 2009, 8, 543. 19525947 10.1038/nmat2442

[52] N. Patel , B. Forbes , S. Eskola , J. Murray , Drug Dev. Ind. Pharm. 2006, 32, 151. 16537196 10.1080/03639040500465991

[53] C. Markopoulos , F. Thoenen , D. Preisig , M. Symillides , M. Vertzoni , N. Parrott , C. Reppas , G. Imanidis , Eur. J. Pharm. Biopharm. 2014, 86, 438. 24184673 10.1016/j.ejpb.2013.10.017

[54] M. Tollemeto , S. Ursulska , P. L. W. Welzen , L. H. E. Thamdrup , A. Malakpour Permlid , Y. Li , G. Soufi , T. Patiño Padial , J. B. Christensen , L. Hagner Nielsen , J. van Hest , A. Boisen , Small 2024, 20, 2403640. 10.1002/smll.202403640 38963162

[55] R. Ebabe Elle , S. Rahmani , C. Lauret , M. Morena , L. P. R. Bidel , A. Boulahtouf , P. Balaguer , J. P. Cristol , J. O. Durand , C. Charnay , E. Badia , Mol. Pharm. 2016, 13, 2647. 27367273 10.1021/acs.molpharmaceut.6b00190

[56] A. Tarantini , R. Lanceleur , A. Mourot , M. T. Lavault , G. Casterou , G. Jarry , K. Hogeveen , V. Toxicity Fessard , Toxicol. In Vitro 2015, 29, 398. 25448807 10.1016/j.tiv.2014.10.023

[57] S. Murugadoss , S. Van Den Brule , F. Brassinne , N. Sebaihi , J. Mejia , S. Lucas , J. Petry , L. Godderis , J. Mast , D. Lison , P. Hoet , Part Fibre Toxicol. 2020, 17, 10.1186/S12989-019-0331-3/TABLES/4. PMC6942297 31900181

[58] F. Wan , M. Herzberg , Z. Huang , T. Hassenkam , H. M. Nielsen , Acta Biomater. 2020, 104, 115. 31945503 10.1016/j.actbio.2020.01.014

[59] Z. Adamczyk , M. Sadowska , P. Żeliszewska , Anal. Chem. 2020, 92, 15087. 32957771 10.1021/acs.analchem.0c03115 PMC7675609

[60] M. Cui , Y. Duan , Y. Ma , K. W. A. Al‐Shwafy , Y. Liu , X. Zhao , R. Huang , W. Qi , Z. He , R. Su , Langmuir 2020, 36, 4503. 32241112 10.1021/acs.langmuir.0c00104

[61] Q. Chen , S. Xu , Q. Liu , J. Masliyah , Z. Xu , Adv. Colloid Interface Sci. 2016, 233, 94. 26546115 10.1016/j.cis.2015.10.004

[62] M. B. Stie , C. Cunha , Z. Huang , J. J. K. Kirkensgaard , P. S. Tuelung , F. Wan , H. M. Nielsen , V. Foderà , S. Rønholt , Sci. Rep. 2024 14:1 2024, 14, 10.1038/s41598-024-72233-1. PMC11393313 39266622

[63] S. Oh , M. Wilcox , J. P. Pearson , S. Borrós , Eur. J. Pharm. Biopharm. 2015, 96, 477. 26272125 10.1016/j.ejpb.2015.08.002

[64] Y. Zhang , S. Li , K. Loch , G. A. Duncan , L. Kaler , R. Pangeni , W. Peng , S. Wang , X. Gong , Q. Xu , ACS Macro Lett. 2023, 12, 446. 36951898 10.1021/acsmacrolett.2c00639

[65] J. T. Huckaby , S. K. Lai , Adv. Drug Deliv. Rev. 2018, 124, 125. 28882703 10.1016/j.addr.2017.08.010

[66] C. Bao , B. Liu , B. Li , J. Chai , L. Zhang , L. Jiao , D. Li , Z. Yu , F. Ren , X. Shi , Y. Li , Nano Lett. 2020, 20, 1352. 31904988 10.1021/acs.nanolett.9b04841

[67] Y. Guo , Y. Ma , X. Chen , M. Li , X. Ma , G. Cheng , C. Xue , Y. Y. Zuo , B. Sun , ACS Nano 2023, 17, 2813. 36719858 10.1021/acsnano.2c11147

[68] M. Tollemeto , P. L. W. Welzen , L. H. E. Thamdrup , Y. Li , T. Patiño Padial , A. Boisen , J. van Hest , Angew. Chem.Int. Ed. 2025, 64, e202419087. 10.1002/anie.202419087 PMC11796314 39446074

[69] M. Tollemeto , I. Badillo‐Ramírez , L. H. E. Thamdrup , Y. Li , M. Ghavami , T. P. Padial , J. B. Christensen , J. van Hest , A. Boisen , Adv. Mater. Interfaces 2024, 11, 2400107.

[70] M. Tollemeto , Z. Huang , J. B. Christensen , H. Mørck Nielsen , S. Rønholt , ACS Appl. Mater. Interfaces 2023, 15, 8810. 10.1021/acsami.2c16502 PMC9951175 36749788

